# Effects of *Astrocaryum aculeatum* Meyer (Tucumã) on Diet-Induced Dyslipidemic Rats

**DOI:** 10.1155/2014/202367

**Published:** 2014-08-03

**Authors:** Geórgia Craveiro Holanda Malveira Maia, Mozer da Silva Campos, Janice Barros-Monteiro, Juan Eduardo Lucas Castillo, Murilo Soares Faleiros, Rejane Souza de Aquino Sales, Denise Moraes Lopes Galeno, Edson Lira, Francisca das Chagas do Amaral Souza, Carmen Ortiz, Luisa Morales, Rosany Piccolotto Carvalho

**Affiliations:** ^1^Programa Multi-Institucional de Pós-graduação em Biotecnologia, Universidade Federal do Amazonas, Avenue General Rodrigo Octávio Jordão Ramos, 3000 Coroado, Manaus, AM, Brazil; ^2^Departamento de Ciências Fisiológicas, Instituto de Ciências Biológicas, Universidade Federal do Amazonas, Avenue General Rodrigo Octávio Jordão Ramos, 3000 Coroado, Manaus, AM, Brazil; ^3^Biochemistry Department, Ponce School of Medicine and Health Sciences, P.O. Box 7004, Ponce, PR 00732-7004, USA; ^4^Fundação de Hematologia e Hemoterapia do Estado do Amazonas (FHEMOAM), Nucleo de Estatística, Avenue Constantino Nery 3240, 69050-001 Manaus, Brazil; ^5^Instituto Nacional de Pesquisas da Amazônia, Coordenação Sociedade Saúde Ambiente-Laboratório de Alimentos, Avenue André Araújo 2.936, Petrópolis, 69067-375 Manaus, AM, Brazil; ^6^Physiology, Pharmacology, and Toxicology Department, Ponce School of Medicine and Health Sciences, P.O. Box 7004, Ponce, PR 00732-7004, USA; ^7^Public Health Department, Ponce School of Medicine and Health Sciences, P.O. Box 7004, Ponce, PR 00732-7004, USA

## Abstract

An *in vivo* study was conducted to assess the effects of the consumption of *Astrocaryum aculeatum Amazon Meyer* (*tucumã*) in the treatment of diet-induced dyslipidemia in sedentary and exercised Wistar rats. With an average weight of 350 grams, 40 male rats were divided into 4 subgroups of 10. The sedentary control group (SCG) was fed with commercial feed, while the sedentary treatment group (STG) was fed with a ration of tucumã. In addition to the sedentary groups, two exercise groups were formed. The Exercised control group (ECG) was fed with commercial food and the exercised treatment group (ETG) was fed with a ration of tucumã. Body weight gain and food intake were monitored during the experiment. Plasma was analyzed for cholesterol, triglycerides, HDL-C, LDL-C, VLDL, total protein, glucose, insulin, and leptin concentrations. Our results show that the ECG group tended to consume more food, while the groups that were fed with tucumã pulp (STG and ETG) presented a greater tendency to gain body mass. ECG group showed a tendency towards a higher concentration of cholesterol in plasma, while STG and ETG presented higher absolute values for triglycerides and VLDL. No hypolipiemic effect was observed related to tucuma ingestion.

## 1. Introduction

Dyslipidemias are characterized by alterations in lipid metabolism, with repercussions on the level of lipoproteins present in the bloodstream. They are directly related to the development of cardiovascular alterations, therefore, promoting the development of chronic cardiovascular and cerebrovascular diseases, such as atherosclerosis, acute myocardial infarction, ischemic disease, and encephalic vascular accident [1–3].

Nontransmissible chronic diseases (NTCDs) are one of the main causes of death worldwide. In Brazil, NTCDs are responsible for approximately 72% of deaths, mostly caused by diseases of the circulatory system (30%), therefore, increasing public health expenses. These diseases cause several alterations in the organism, leading to premature death or even disability, eventually, limiting the individual's working opportunities and consequently increasing social care expenditure [4, 5]. Risk factors triggering NTCDs include tobacco and alcohol consumption, sedentary lifestyle, and unhealthy food habits. It is crucial for the Brazilian public health system to develop strategies aiming to decrease the risk of these diseases in the population and to incorporate healthcare services targeting these diseases [6, 7].

Limited exercise and inadequate food habits such as a high-fat and low-fiber diet can lead to the accumulation of adipose tissue and the consequent development of obesity. This triggers alterations in the lipid metabolism and accelerates the onset of hypercholesterolemia and triglyceridemia among other metabolic alterations leading to the development of coronary diseases [8, 9]. Most carriers of hypercholesterolemia do not show signs or symptoms that can be directly attributed to a lipid alteration, and the diagnosis is based almost solely on a serological analysis of lipids in plasma. However, some patients with more severe hyperlipidemias, usually of hereditary nature, may present clinical manifestations such as fat accumulation in several tissues (i.e., eyes, skin, tendons, and nervous system) that are externally visible, which can lead to suspect a lipid alteration even before serological determination [10].

According to Sposito et al., previous studies have demonstrated that a reduction of cholesterol levels can decrease the risk of cardiovascular events, the incidence of cardiac ischemic disease (by 25%–60%), and the risk of death by 30%. Cholesterol reduction can also decrease other morbid effects such as angina and cerebrovascular accidents among other conditions [3]. Soares and coworkers found that a 1% reduction in serum cholesterol levels results in a 2% reduction in the risk of coronary diseases [11]. Given the crucial role of cholesterol in these morbidities, it is vital to find alternatives to allow its control and treatment.

Evidence shows that pharmacological treatment can reduce the risk factors related to dyslipidemia. However, they can be prevented with lifestyle changes, including physical exercise in combination with nutritional therapy [3]. Therefore, preventive measures that decrease fat accumulation like changes in diet and physical activity can contribute to balance the blood lipoprotein concentrations. Moreover, the beneficial effects of these measures have been well established for normolipidemic and dyslipidemic patients alike [12].

It is known that the intake of fiber, especially insoluble ones, as well as the moderate consumption of unsaturated fatty acids is beneficial in the reduction of lipids in plasma. Fibers reduce the absorption of intestinal cholesterol in food, regularizing the enteric food flow and promoting glycemic and cholesterolemic control [13]. In addition to these physiological effects, the ingestion of polyunsaturated or monounsaturated fatty acids reduces total blood cholesterol, as well as low density lipoprotein C (LDL-C) and triglycerides that can help to avoid lipid oxidation [3].

Foods with favorable characteristics for the treatment of diseases are described as functional foods. The tucumã (*Astrocaryum aculeatum *Meyer) palm can be found in degraded environments among secondary vegetation in the Central and Western Amazonian ecosystem. Its fruit is widely consumed by the Amazonian population* in natura*, in sandwiches and tapioca, desserts, and ice cream. Indians apply the oil extracted from the pulp of the fruit and the seeds over their body and hair [14].

Tucumã is a smooth drupe with an epicarp and mesocarp that can vary greatly in color (yellowish to dark orange and red, or whitish), shape (oval, spherical), mesocarp thickness (2.8 to 10 mm), endocarp, and seed type. The consistency of the fruit is mucilaginous, oily, and sweet to the taste with a characteristic flavor. Its weight varies from 20 to 100 g, of which 22.4% is edible [15–17]. As to nutritional composition, the high crude fiber content stands out. Furthermore, in some fruits of the same genus, there are unsaturated fatty acids which are beneficial if taken moderately [18].

According to Ferreira et al., the Palm tucumã (*Astrocaryum vulgare Mart*) is composed of fatty acids that may induce a reduction of cholesterol in plasma, triglycerides, and LDL-cholesterol, as well as an increase of the HDL-c concentration [18]. This could be due to the fact that, among all the fatty acids that constitute its crude oil, a concentration of 68% monounsaturated fatty acids and only 29% saturated fatty acids is found. These data were also evident in the studies by Guedes [19].

From the centesimal composition analysis of the Palm tucumã (*Astrocaryum vulgare *Mart), and according to studies by Aguiar et al., and Leitão [17, 20], it can be assumed that the key components are present in similar concentrations, as shown in Table 1.

Apart from the beneficial fiber concentration, Guedes demonstrated that another type of tucumã (*Astrocaryum vulgare* Mart) of the same genus as the one studied is composed of 29.6 g of palmitic acid, 3.0 g of stearic acid, 58.5 g of oleic acid, 3.8 g of linoleic acid, and 5.5 g of linolenic acid [19]. This suggests that the intake of the tucumã (*Astrocaryum aculeatum Meyer*) may be beneficial, given that unsaturated fatty acids, when taken moderately, may be favorable to patients with dyslipidemias.

Since there is no previous research on the use of the Amazonas' tucumã as a functional food for the control or reduction of dyslipidemia, the relevance of our project becomes apparent. Hence, the objective of this study is to assess the effects of tucumã fruit (*Astrocaryum aculeatum* Meyer) intake in the treatment of diet-induced dyslipidemia in sedentary and exercised Wistar rats.

## 2. Material and Methods

### 2.1. Animals

Forty male rats of the Wistar strain, obtained from the Animal House of the Amazonas Federal University (UFAM), weighting between 250 and 350 grams, were used to perform the study. They were kept under a 12-hour light-dark cycle, with free (*ad libitum*) access to food and water under adequate light and temperature conditions. The experimental protocol was approved by the Animal Experimentation Ethics Committee of the same university, protocol number 020/2011.

### 2.2. Experimental Diets

All diets contained Labina commercial feed for laboratory animals, produced by Agribrands do Brasil Ltda of Paulínia, SP. The experimental protocol was approved by the Animal Experimentation Ethcontrol groups. In the first part of the experiment, a hyperlipidic food portion was prepared using pork lard to induce dyslipidemia. It was composed of 36% of Sadia lard, 28% of Synth casein, and 36% of commercial feed [21].

**Figure 1:**
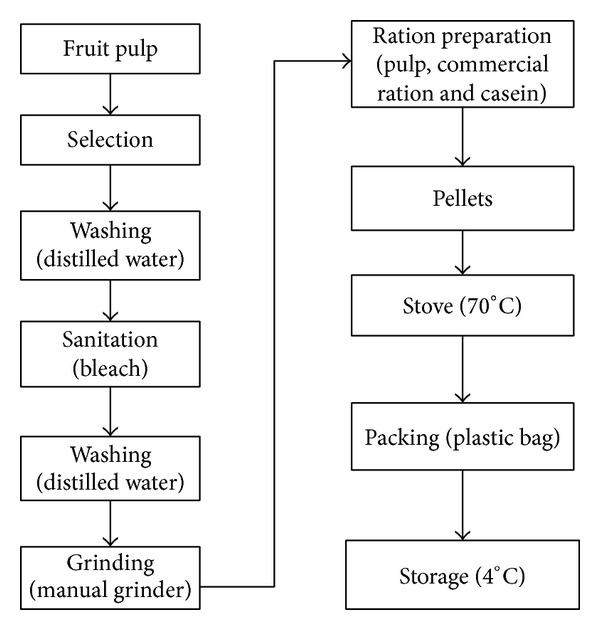
Fruit processing and ration preparation.

The treatment portion was composed of 12% of casein, 28% of commercial feed, and 60% of tucumã pulp from a farm located in the Municipality of Rio Preto da Eva (AM), and posteriorly processed at UFAM according to the methodology proposed by Yuyama et al. with some modifications (Figure 1) [14].

### 2.3. Determination of Proximate Composition of Tucumã Based Diets

To determine the fruit pulp concentration necessary for the treatment, rations with different concentrations of tucumã pulp (20%, 30%, and 60%) were prepared, followed by physicochemical analysis at the Fish Technology Laboratory at UFAM. Humidity, protein, fat, ash, crude fiber, and carbohydrate content were analyzed according to procedures of the Fish Technology Laboratory. In addition, the proportions of soluble and insoluble fibers of the rations with different fruit concentrations were also investigated at the National Research Institute of Amazonia, according to the methodology adopted by the institute.

### 2.4. Experimental Groups

Four groups of 10 animals were used in the study. Each group was designated according to the treatment used and the amount of physical activity as sedentary control group (SCG), which were fed commercial feed and did not perform physical activity, exercised control group (ECG), which were fed commercial feed and exercised, sedentary tucumã group (STG), which were fed a portion of tucumã pulp and did not perform physical activity, and exercised tucumã group (ETG), which were fed a portion of tucumã pulp and exercised. Food intake was monitored over time and body mass gain was determined.

### 2.5. Physical Activity Protocol

Animals were submitted to an aerobic exercise program. The protocol consisted of a daily session of swimming for sixty minutes, three days a week, during eight weeks, in a plastic tank with a height of 120 cm and a diameter of 80 cm filled with water at 30°C. During the sessions, the animals had a resistance equivalent to 3 to 5% of their body weight attached to the tail as described by Rodrigues and Zambon et al., with minor modifications [21, 22].

### 2.6. Determination of Selected Plasma Biomolecules

Plasma samples were collected at the beginning, after 30 days, and on the last day of the experiment. Blood was collected by punction of the animals' tails, stocked in 1.5 mL heparinized microtubes, and centrifuged at 3000 rpm for 5 minutes for plasma separation and immediate analysis of the concentrations of cholesterol, triglycerides, HDL-C, LDL-C, VLDL, total protein, glucose, insulin, and leptin. These analyses were performed using a colorimetric enzymatic method using an In Vitro Diagnno Dia S/A (Itabira, MG) commercial kit [23].

### 2.7. Statistical Analysis

To assess the influence of tucumã and physical exercise in our study, variance analysis (ANOVA) and Tukey's test were used to establish statistical differences among the groups. The difference was considered significant when *P* < 0.05. Results were expressed as average ± standard deviation.

## 3. Results

After conducting this study for 90 consecutive days, plasma and proximate composition analyses were performed, and food intake and body mass gain were assessed for the different experimental groups.

### 3.1. Proximate Composition of Food Portion with Increasing Concentration of Tucumã

The three samples with different concentrations of tucumã showed similar physicochemical properties. However, a higher concentration of fat was reported for the 60% sample, which is twice of the concentration in the 30% sample, and three times higher than that of the 20% sample. In addition, a higher proportion of carbohydrates was found in the 20% sample and a much lower proportion in the 60% sample. As to fibers, the concentration was similar among the three samples (Table 2).

### 3.2. Food Intake and Body Mass Gain

**Figure 2:**
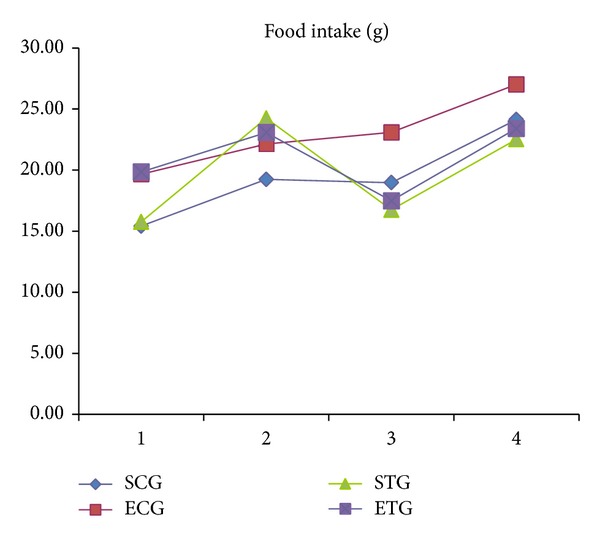
Average values of food intake (g) among the studied groups throughout the experimental period. Note: SCG: sedentary control group; ECG: exercised control group; STG: sedentary tucumã group; ETG: exercised tucumã group. *P* value < 0.001 for all groups.

Figure 2 illustrates food intake and shows no statistically significant difference among the studied groups (*P* = 0.495). In addition, the results show a tendency of higher food intake in the ECG when compared to the other groups.

**Figure 3:**
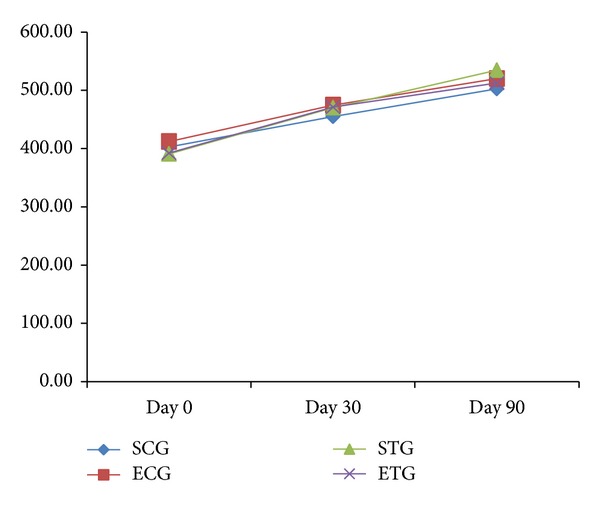
Average body mass gain values (g) of the groups evaluated at days 0, 30, and 90. Different letters in superscript denote significant difference for *P* < 0.05. Note: SCG: sedentary control group; ECG: exercised control group; STG: sedentary tucumã group; ETG: exercised tucumã group.

Figure 3 shows the average body mass gain in the groups under study. In the SCG, there was a statistically significant difference between body mass gain only between day 0 and day 90. In the ECG, there was a statistically significant difference among the three time intervals of the experiment. STG and ETG did not show significant body mass differences between day 30 and day 90. However, these two time points were significantly different from the body mass at day 0. The descriptive analysis demonstrated that the groups that consumed the diet with tucumã fruit showed a higher tendency to gain body mass, whereas animals belonging to the exercise groups (ECG and ETG) showed a reduced tendency to gain body mass.

### 3.3. Biochemical Analysis

#### 3.3.1. Lipid Profile

The lipids profile of the groups under study was analyzed separately at days 0, 30, and 90 (Table 3). At the beginning of the experiments (day 0), there was no statistically significant difference among the groups in relation to cholesterol levels, which demonstrates the homogeneity of this parameter among the experimental groups. At this time point, triglycerides and VLDL of ETG and SCG were statistically different, ETG presenting higher concentration of these lipids.

After the induction period on day 30, it can be seen that there was no statistically significant difference among groups for any of the lipids evaluated. Although ECG showed a tendency for higher concentration of cholesterol in plasma, STG and ETG presented higher absolute values for triglycerides and VLDL.

At the end of the experiment (day 90), we found that the variance analysis of cholesterol levels did not reveal a statistically significant difference among the groups. However, it was observed that the groups that consumed tucumã had a higher concentration of this lipid. On the other hand, the plasma triglyceride analysis revealed that the SCG had much higher values in comparison with ECG, STG, and ETG. Furthermore, descriptive statistics analysis showed that groups that exercised presented lower triglyceride levels in comparison to sedentary groups. VLDL was shown to be significantly different between SCG and ETG. Exercised groups also presented lower values in comparison to the sedentary ones.

Among the groups that exercised (ECG and ETG), the group consuming tucumã presented a reduction of the lipid concentration in plasma.

### 3.4. Protein, Glucose, Insulin, and Leptin

Table 4 shows that the values related to protein and insulin concentration were not significantly different among the experimental groups. However, concentration of glucose in plasma was statistically different among the control groups (SCG and ECG). Leptin concentration in plasma was found to be different between ECG and STG.

## 4. Discussion

This study aimed to assess the effect of tucumã (*Astrocaryum aculeatum* Meyer) intake in lipid metabolism. Since it is a regional fruit from Amazonia and complies with the characteristics established by the Dyslipidemia Brazilian Society, tucumã should be considered a functional food due to its medicinal properties that can promote benefits to health [3]. Functional foods possess beneficial properties in addition to its basic nutritional properties, presented in the form of common foods. They are part of conventional diets but they show the capacity of regulating body functions and aiding in the protection against diseases such as hypertension, diabetes, cancer, osteoporosis, and coronaropathy [24, 25].

It can be seen that food intake (Figure 2) among the groups was probably regulated by the ingestion of calories, because although they are present* ad libitum* there was not a higher intake by any experimental group. Our data agree with the study of Rocha et al., who also recorded a proportional food intake among all studied groups [26]. An increase in body mass was observed after the beginning of the treatment in all animals (Figure 3). Hence, the significant reduction among exercised groups in relation to sedentary groups and among groups that ingested tucumã was not apparent in this study. These same results were found in studies by Bell et al. and Pellizzon et al., who recorded weight gain by animals at the end of the experiment even in those that exercised and those that had a diet with polyunsaturated fatty acids [27, 28].

Although there was no significant difference in food intake and body mass among all groups, STG showed a greater tendency to body mass gain (Figure 3). This could be due to the addition of tucumã to their diet. Taking into consideration the high energy value of tucumã and the absence of physical activity in this group, we could provide a possible explanation for the accumulation of fat and body mass gain in these animals.

Using an animal model, Franco et al. demonstrated that the consumption of large quantities of saturated fatty acids promotes a greater accumulation of fat and consequently a greater body mass gain, when compared to the ingestion of a diet rich in polyunsaturated and monounsaturated fatty acids [29]. In contrast, Lombardo and Chicco relate a greater weight gain and obesity in rats fed on a diet with polyunsaturated fatty acids when compared to saturated fatty acids [30]. However, since the analysis of the type of fatty acids that compose the tucumã (*Astrocaryum aculeatum* Meyer) was not performed, it cannot be asserted exactly which one was responsible for the body mass increase in the STG.

Therefore, the lesser tendency to body mass gain of ECG and ETG (Figure 3) is probably related to the performance of light physical exercise, such as swimming, and not to the intake of tucumã, since it is already established in the literature that aerobic exercises lead to increased mitochondrial function, favoring fatty acid oxidation. Furthermore, these exercises facilitate fat mobilization, stimulating the action of the hormone-sensitive lipase [29].

In this study, as in those by Curi et al. and Morais et al., hyperglycemia was noticed (Table 4) in all groups, even in those subjected to physical activity [31, 32]. Since all animals showed an increase in body mass, a tendency to develop obesity and probably a metabolic alteration (i.e., resistance to insulin) could be established.

It is known that when physical exercise is performed, the available fat is used preferentially for energy production (oxidation), decreasing glycogen degradation and glucose oxidation [29]. When the amount of fat increases, the organism can develop resistance to the action of insulin, thus, favoring an increase in the concentration of glucose in plasma. Previous research has shown that obesity, with the accumulation of visceral fat, is one of the main drivers for the development of this resistance. These alterations were detected in our study, allowing us to establish an association between these factors and the development of hyperglycemia in the experimental groups [33–35].

However, insulin and leptin (a hormone that participates in energy balance regulation by reducing food ingestion and increasing energy expenditure) both remained unaltered in relation to plasma concentration (Table 4). This could be due to the short time interval used for this experiment. We believe it was not enough to promote that alteration. When obesity develops, a decreased sensitivity to leptin occurs in parallel leading to a lesser activity, which may justify the body mass increase and reduced activity [35, 36].

The results obtained for protein in plasma (Table 4) did not express alteration of basal values when compared to studies performed to evaluate biochemical parameters of rats of the same strain [37]. As to the lipidogram, it can be seen that STG and ETG, both fed with tucumã, showed a relatively higher increase in total cholesterol when compared with SCG and ECG (Table 2).

The absence of hypocholesterolemic effect was also found in the study by Rocha et al. with the use of fruta-de-lobo (*Solanum lycocarpum *A. St.-Hit) that, even after 42 days of experimentation, did not reduce total cholesterol levels [26]. However, other studies demonstrate the inhibitory effect of the HMG CoA reductase enzyme derived from the consumption of vegetables like eggplant (*Solanum melongena*), (*Solanum gilo*), onion (*Allium cepa*), and carrot (*Daucus carota*) [38]. Other fruits have been found to promote the reduction of triglycerides and total cholesterol through other metabolic pathways, due to their physicochemical characteristics, such as the camucamu (*Myrciaria dubia*) [39]. Therefore, we can state that, due to its physicochemical characteristics, the ingestion of tucumã in high concentrations promotes metabolic alterations favorable to the development of dyslipidemia. So, we expected that given the fatty acid composition of the Pará tucumã, the Amazonas tucumã, which is a different species of the same genus, could also reduce lipids in plasma. However, this assumption was not confirmed, which allows us to suggest that the Amazonas tucumã has a different fatty acid composition that can lead to the development of obesity, resistance to insulin, and dyslipidemia. Therefore, nutrient unbalance in the diet is associated with metabolic alterations, such as overweight, hyperlipidemia with high levels of triglycerides in plasma, low levels of HDL, resistance to insulin, and retardation in glucose clearance, probably due to an increase of free circulating fatty acids, related to a reduction in life expectancy [40–42].

Some of these alterations, like body weight increase (Figure 3), leading to the development of obesity, as well as hyperlipidemia (Table 3) and hyperglycemia (Table 4), were found in this experiment, mainly in sedentary groups (SCG and STG), but markedly in the sedentary group fed with tucumã (STG).

## 5. Conclusion

An adequate diet in combination with changes in lifestyle can promote positive alterations in lipid metabolism, as well as the balance of organic functions and consequent reduction of risk factors for the development of hypercholesterolemia and cardiovascular diseases. This study showed that the tucumã fruit (*Astrocaryum aculeatum *Meyer) does not have a hypolipidemic effect, since animals presented high lipid concentrations. Furthermore, it is apparent that low intensity aerobic physical activity (i.e., swimming) exerts a positive effect in the lipid profile of Wistar rats, since it was observed that exercised animals showed reduction in some biochemical parameters. However, we conclude that the duration of the exercise was not sufficient, since it promoted slightly significant alterations.

## Figures and Tables

**Table 1:** Proximate composition of the tucumã fruit *in natura*.

Determination	Aguiar et al. (1980) [17] *n* =	Leitão (2008) [20] *n* =	Ferreira et al. (2008) [18] *n* =
Humidity (%)	38.5	50.25 ± 11.49*	44.90 ± 0.30*
Protein (g)	5.5	3.39 ± 1.08*	3.54 ± 0.07*
Fat (g)	47.2	25.19 ± 10.91*	40.49 ± 0.54*
Crude fiber (g)	19.2	28.71 ± 1.86*	10.93 ± 0.10*
Ash (g)	2.0	1.23 ± 0.27*	2.53 ± 0.05*
Total carbohydrates (g)	6.8	19.25 ± 1.69*	8.54 ± 0.61*
Energy (Kcal/100 g)	474.0	320.0 ± 98.75*	412.73 ± 2.12*

*Mean ± SD.

**Table 2:** Physicochemical characterization of the dry food portion based on tucumã pulp concentration.

Tucumã pulp (%)	Humidity (%)	Ash (%)	Protein (%)	Lipids (%)	Carbohydrates (%)	Fibers (%)
20%	7.97 ± 0.15*	7.41 ± 0.04*	19.39 ± 1.14*	4.71 ± 0.85*	60.52 ± 0.92*	2.23 ± 0.09*
30%	7.53 ± 0.58*	7.08 ± 0.11*	21.67 ± 0.19*	6.31 ± 0.51*	58.41 ± 1.14*	2.33 ± 0.08*
60%	8.47 ± 0.07*	5.27 ± 0.07*	27.10 ± 0.65*	14.71 ± 0.94*	44.45 ± 0.61*	2.49 ± 0.19*

Asterisks (∗) denote the difference with the control group.

Values are presented as average values ± S.E., where *P* ≤ 0.05. Note: S.E.: standard error.

**Table 3:** Lipid profile of the groups under study at different time points (days 0, 30, and 90).

GROUPS	Day 0			Day 30			Day 90		
	Chol.	TG	VLDL	Chol.	TG	VLDL	Chol.	TG	VLDL
SCG	56.72 ± 4.47^a^ *n* = 9	52.40 ± 3.96^a^ *n* = 8	10.08 ± 0.81^a^ *n* = 9	82.09 ± 12.70^a^ *n* = 8	72.02 ± 13.94^a^ *n* = 8	18.62 ± 4.67^a^ *n* = 9	186.61 ± 13.17^a^ *n* = 8	174.90 ± 17.31^a^ *n* = 6	24.31 ± 2.70^a^ *n* = 9

ECG	70.87 ± 4.60^a^ *n* = 9	83.35 ± 13.98^a,b^ *n* = 10	16.67 ± 2.80^a,b^ *n* = 10	122.26 ± 12.55^a^ *n* = 9	96.85 ± 15.74^a^ *n* = 10	19.37 ± 3.15^a^ *n* = 10	219.44 ± 16.91^a^ *n* = 7	80.18 ± 14.31^b^ *n* = 10	16.04 ± 2.86^a,b^ *n* = 10

STG	71.77 ± 2.95^a^ *n* = 10	75.78 ± 12.17^a,b^ *n* = 9	16.74 ± 2.69^a,b^ *n* = 10	101.84 ± 8.50^a^ *n* = 10	114.14 ± 24.25^a^ *n* = 9	26.77 ± 5.86^a,b^ *n* = 10	260.79 ± 22.93^a^ *n* = 8	106.74 ± 18.82^b^ *n* = 9	24.04 ± 4.31^a,b^ *n* = 10

ETG	56.72 ± 4.47^a^ *n* = 9	52.40 ± 3.96^a^ *n* = 8	10.08 ± 0.81^a^ *n* = 9	82.09 ± 12.70^a^ *n* = 8	72.02 ± 13.94^a^ *n* = 8	18.62 ± 4.67^a^ *n* = 9	186.61 ± 13.17^a^ *n* = 8	174.90 ± 17.31^a^ *n* = 6	24.31 ± 2.70^a^ *n* = 9

*P* value among groups	70.87 ± 4.60^a^ *n* = 9	83.35 ±13.98^a,b^ *n* = 10	16.67 ± 2.80^a,b^ *n* = 10	122.26 ± 12.55^a^ *n* = 9	96.85 ± 15.74^a^ *n* = 10	19.37 ± 3.15^a^ *n* = 10	219.44 ± 16.91^a^ *n* = 7	80.18 ± 14.31^b^ *n* = 10	16.04 ± 2.86^a,b^ *n* = 10

Values shown represent the average ± S.E. expressed in mg/dL. ^a,b^Values followed by different letters in superscript present significant differences among the different time points for *P* < 0.05. Note: SCG: sedentary control group; ECG: exercise control group; STG: sedentary tucumã group; ETG: exercise tucumã group; S.E.: standard error; *n*: number of animals evaluated per group.

**Table 4:** Total protein, glucose, insulin, and leptin concentrations of the different groups on day 90.

Groups	SCG	ECG	STG	ETG	*P* value
Protein (g/dL)	6.35 ± 0.45^a^ *n* = 9	6.83 ± 0.37^a^ *n* = 10	6.15 ± 0.36^a^ *n* = 10	5.98 ± 0.46^a^ *n* = 9	<0.500

Insulin (ng/dL)	3.17 ± 0.39^a^ *n* = 9	2.30 ± 0.26^a^ *n* = 7	2.43 ± 0.53^a^ *n* = 9	2.47 ± 0.55^a^ *n* = 9	<0.516

Glucose (mg/dL)	171.91 ± 1.91^a^ *n* = 9	162.36 ± 3.27^b^ *n* = 10	170.46 ± 1.39^a,b^ *n* = 10	165.58 ± 3.51^a,b^ *n* = 9	<0.040

Leptin (ng/dL)	7.70 ± 0.94^a,b^ *n* = 9	5.15 ± 0.44^a^ *n* = 9	9.86 ± 1.11^b^ *n* = 9	8.43 ± 1.19^a,b^ *n* = 9	<0.007

^a,b^Values (average ± S.E.) followed by different letters in superscript present significant differences for *P* < 0.05. Note: SCG: sedentary control group; ECG: exercise control group; STG: sedentary tucumã group; ETG: exercise tucumã group; S.E.: standard error; *n*: number of animals evaluated per group.
